# Inflammation in Diabetic Kidney Disease Is Linked to Gut Dysbiosis and Metabolite Imbalance

**DOI:** 10.1111/1753-0407.70175

**Published:** 2025-12-14

**Authors:** Xueting Zheng, Xiaoyan Luo, Yi Zhang, Zhiyan Zou, Jiayi Yang, Huan Liu, Zhou Lu, Fangfang Cao, Xilian Wang, Xinyun Ge, Xiaoan Li, Jiali Wang

**Affiliations:** ^1^ NHC Key Laboratory of Nuclear Technology Medical Transformation, Mianyang Central Hospital Mianyang China; ^2^ North Sichuan Medical College Nanchong China; ^3^ Department of Gastroenterology Mianyang Central Hospital Mianyang China; ^4^ The Affiliated Hospital of Southwest Medical University Luzhou China; ^5^ Department of Pediatrics Mianyang Central Hospital Mianyang China; ^6^ Department of Nephrology Mianyang Central Hospital Mianyang China; ^7^ Southwest University of Science and Technology Mianyang China

**Keywords:** diabetic kidney disease, gut dysbiosis, inflammation, metabolite imbalance, molecular mechanisms

## Abstract

**Background:**

Diabetic kidney disease (DKD) is characterized by a sustained pro‐inflammatory response of the immune system, which leads to renal failure progression and related complications. Emerging evidence suggests that gut microbiota dysregulation may be a pathogenic mediator in DKD, while mechanisms remain unclear. This study aimed to identify differences in the gut microbiota of the DKD group and healthy controls (HC).

**Methods:**

Gut microbiota composition was determined using shotgun metagenomic sequencing on fecal samples; serum cytokines were measured via ELISA, immune phenotypes were detected using flow cytometry.

**Results:**

Significant differences in gut microbiota diversity and richness were observed between patients with DKD and HC, with higher abundances of Enterobacteriaceae, *Serratia*, and *Shigella* in the DKD group than in the HC group. Additionally, CD3+ (especially CD4+) T cells were significantly higher in the renal tissue of the DKD group than the HC group. Flow cytometry identified significantly higher circulating levels of NKT cells and CD8+ T cells and lymphocyte ratio in HC than in DKD. CD4+ cells, CD4+ TCM cells, CD8+ TCM cells, and the CD4+/CD8+ cell ratio were significantly higher in the DKD group than in the HC group, as were levels of pro‐inflammatory mediators, including IL‐6, TNF‐α, and sCD14, and expression of the gut barrier dysfunction marker ZO‐1.

**Conclusions:**

Gut barrier dysfunction and gut microbiota imbalance may mediate the pro‐inflammatory immune phenotype observed in patients with DKD and thereby contribute to DKD progression. These findings underscore the important role of the microbiota–immune axis in the development of DKD.

## Highlights

Gut barrier dysfunction and gut microbiota imbalance may mediate the pro‐inflammatory immune phenotype observed in patients with DKD and thereby contribute to DKD progression. These findings underscore the important role of the microbiota–immune axis in the development of DKD.

## Graphical abstract

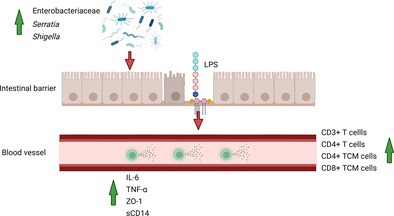

## 1 Introduction

Diabetic kidney disease (DKD) is the main cause of chronic kidney disease and ultimately leads to irreversible renal failure; increasing evidence has identified gut microbiota dysbiosis as both a prognostic biomarker and pathogenic mediator in DKD pathophysiology [1, 2]. Abnormal metabolites derived from the gut microbiota may compromise intestinal barrier integrity, exacerbate inflammation, and activate both the immune and renin–angiotensin systems [3, 4].

The gut microbiota is considered the largest immune organ in the human body [5]. Various investigations have demonstrated that the perturbation of the gut microbiota and its associated metabolites potentially underlie the etiology and progression of DKD. This phenomenon may transpire through perturbation of both the innate and the adaptive immunity, leading to a burdensome allostatic load on the body and ultimately culminating in the development of DKD [6, 7]. We aim to delve into the intricate interplay between the gut microbiota, its metabolites, and the immune system in the context of DKD, in order to provide new targets and clinical insights for the diagnosis and treatment of DKD.

In this study, we recruited patients with DKD and age‐matched healthy controls (HC). We identify a DKD‐specific gut microbial composition, as well as altered microbial metabolism of nutrients and corresponding effects on inflammation and immune cell dysregulation in patients with DKD. The early detectability of these dysbiosis‐driven immunological changes in patients with DKD highlights the potential of microbiota‐targeting therapies to improve the prognosis of patients with DKD.

## 2 Patients and Methods

### 2.1 Patient Inclusion and Exclusion Criteria

A total of 59 patients were considered to be eligible and were enrolled in the study. The diagnosis and classification of diabetes mellitus was based on the criteria of the American Diabetes Association (ADA) [8]. DKD was diagnosed with the low estimated glomerular filtration rate (eGFR) (< 60 mL/min/1.73 m^2^) or albuminuria (urinary albumin‐to‐creatinine ratio [ACR] ≥ 30 mg/g) in type 2 diabetes mellitus (T2DM) patients [9]. The eGFR was calculated using the Chronic Kidney Disease Epidemiology Collaboration (CKD‐EPI) equation for standardized creatinine [10].

The inclusion criteria were patients (1) aged ≥ 18 years, (2) with a diagnosis of T2DM, (3) with an eGFR > 15 mL/min/1.73 m^2^ [11]. The exclusion criteria were subjects without T2DM, a history of kidney transplantation or incomplete clinical data, acute or chronic inflammatory diseases, fever, chronic liver disease, inflammatory bowel disease, or other gastrointestinal disorders such as constipation, diarrhea, short bowel syndrome. Patients with antibiotic prophylaxis or treatment within the 4 weeks prior to recruitment were excluded. All procedures were conducted after obtaining informed consent from each participant. This study protocol was reviewed and approved by the Biomedical Ethics Committee of Mianyang Central Hospital (S20240323‐01).

### 2.2 Clinical Assessment, Biobanking and Routine Laboratory Measurements

The following baseline clinical characteristics were collected: age, gender, serum albumin, hemoglobin, blood urea nitrogen (BUN), serum creatinine, low‐density lipoprotein cholesterol (LDL‐C), high‐density lipoprotein cholesterol (HDL‐C), total cholesterol, triglyceride, urinary albumin‐to‐creatinine ratio (ACR), and eGFR, which was calculated using the equation of the Chronic Kidney Disease–Epidemiology Collaboration.

Heparinized blood specimens were collected as part of routine laboratory sampling and used to measure creatinine, urea, hemoglobin (HBG), albumin (ALB), total cholesterol, triglyceride, LDL‐C, HDL‐C. The serum was stored at −80°C for further use. Tumor necrosis factor‐a (TNF‐α), interleukin‐6 (IL‐6), zona occludens‐1 (ZO‐1), and soluble CD14 (sCD14) were analyzed in patients' serum using the human TNF‐α, IL‐6, ZO‐1, and sCD14 ELISA kit (Elabscience, China), according to the manufacturer's protocol. All samples were analyzed on a single plate on a microplate reader (Agilent, USA).

Stool specimens (2–4 g) were collected and stored for 24 h at 4°C–8°C maximum and then transferred to the study center for freezing at −80°C. Patients were provided with detailed information about the collection, storage, and transportation of stool specimens.

### 2.3 Flow Cytometry Analysis

Immune cells in fresh peripheral blood samples were analyzed using multicolor flow cytometry. For cell surface staining, 1 × 10^6^ cells per tube were stained for surface markers with antibodies for 30 min at 4°C in the dark. For intracellular staining, the cells were first stained for surface markers before being fixed/permeabilized and stained according to the manufacturer's instructions. The antibodies used in this study are listed in the Supporting Information. A Celesta flow cytometer (BD Immunocytometry Systems, San Diego, CA, USA) was used to acquire the data, which were analyzed using FlowJo software vX.0.7 (Treestar, San Carlos, CA, USA).

### 2.4 Immunohistochemistry

Immunohistochemistry was conducted in accordance with established protocols. In this experiment, sections were treated with hydrogen peroxide for 10 min to block endogenous peroxidase activity. Following this, sections were washed for 15 min three times with Tris‐buffered saline and Tween (TBS‐T). Subsequently, sections were blocked with UltraVision protein blocking agent (TL‐060‐QHL; Thermo Fisher Scientific) at room temperature for 10 min. The slides were incubated with antibodies targeting CD3 (1:1000; MAB‐0740; Mxb biotechnology), CD4 (1:1000; ab288724; Abcam), and CD8 (1:1000; ZA‐0508; Zsgb‐bio) in a solution containing 3% bovine serum albumin at 4°C overnight. Subsequently, slides were washed for 15 min three times with TBS‐T at room temperature. Slides were then subjected to a 10‐min incubation with the primary antibody. Thereafter, they were rinsed for 15 min three times with TBS‐T. Afterwards, slides were treated with horseradish peroxidase polymer Quanto for 10 min. Finally, slides were washed with TBS‐T at room temperature for 15 min three times. Slides were subjected to treatment with 3,3‐diaminobenzidine, followed by cessation of the reaction using water. Subsequently, sections were exposed to hematoxylin for 3 min at ambient temperature. The kit was utilized in accordance with the manufacturer's instructions for Masson's trichrome staining. Following this, mounting medium was applied to the slides and coverslips were installed. Once the mounting medium had solidified, sections were scanned using the Nikon Imaging system (DS‐U3; Nikon).

### 2.5 Metagenomic Sequencing of Gut Microbiomes

Fecal samples were collected from 31 patients with DKD and 28 HC. The extracted DNA was randomly fragmented into smaller fragments, and sequencing libraries were subsequently prepared. The adapter‐ligated fragments were then PCR‐amplified, size‐selected, and purified. Library concentration was determined using a Qubit fluorometer and real‐time PCR, while size distribution was assessed using a bioanalyzer. The quantified libraries were pooled and sequenced on an Illumina platform, according to their effective concentrations and required data volume. Raw sequencing data were preprocessed using Readfq software to obtain clean data for further analysis. Clean data were assembled using MEGAHIT software. Open reading frames of scaffolds were predicted using MetaGeneMark with default parameters. DIAMOND software was employed to align unigene sequences with those from bacteria, fungi, archaea, and viruses in the NCBI NR database, and the NR database was used to align unigenes with functional databases.

### 2.6 Statistical Analyses

Detailed methods for statistical analyses of microbiome populations are presented in the preceding section. In addition, Spearman correlation analyses were performed to estimate the correlation between inflammatory markers and intestinal leakage markers. Data analyses were conducted using SPSS version 20.0 (SPSS Inc., Chicago, IL, USA) and GraphPad Prism 9.3.1 (GraphPad Software, La Jolla, CA, USA). Comparisons between two groups were made using unpaired *t*‐tests. For continuous variables, data are presented as the mean ± standard deviation or median with range. Differences in means among groups were evaluated using one‐way ANOVAs, followed by least significant difference tests for multiple comparisons. Chi‐squared or Fisher's exact tests were used to compare categorical variables. Statistical significance was determined as follows: *p < 0.05 and **p < 0.01 when comparing the DKD and HC groups.

## 3 Results

### 3.1 Clinical Characteristics of the Total Population

The baseline data of the 59 patients enrolled in this study is shown in Table 1. The study included 47.46% (28) individuals in the HC group and 52.54% (31) in the DKD group. The mean age was 60 years old, and 55.93% of the subjects were male. The mean serum albumin level was 42.96 ± 6.42 g/L, and the mean hemoglobin level was 128.08 ± 24.44 g/L. The mean BUN level was 9.03 ± 5.10 mmol/L. The mean serum creatinine level was 1.45 ± 1.07 mg/dL. The mean eGFR was 55.23 ± 26.05 mL/min/1.73 m^2^. The mean triglyceride level was 1.77 ± 0.85 mmol/L. The mean cholesterol level was 4.80 ± 1.34 mmol/L. The mean LDL‐C level was 2.77 ± 1.10 mmol/L, and the mean HDL‐C level was 1.41 ± 0.40 mmol/L. The serum albumin, hemoglobin, BUN, serum creatinine, e‐GFR, triglyceride, LDL‐C and HDL‐C levels showed statistical significance between the two groups (Table 1).

**TABLE 1:** Clinical characteristics of the total included population.

	Total (*n* = 59)	HC (*n* = 28)	DKD (*n* = 31)	*p*
Age (years)	60.71 ± 8.98	60.11 ± 7.02	61.26 ± 10.54	0.627
Male (%)	33 (55.93%)	14 (50.00%)	19 (61.291%)	0.383
Serum albumin (g/L)	42.96 ± 6.42	47.65 ± 3.24	38.79 ± 5.69	< 0.001
Hemoglobin (g/L)	128.08 ± 24.44	145.04 ± 13.34	112.77 ± 22.00	< 0.001
Serum creatinine (mg/dL)	1.45 ± 1.07	0.75 ± 0.16	2.09 ± 1.14	< 0.001
BUN (mmol/L)	9.03 ± 5.10	5.33 ± 0.93	12.38 ± 5.00	< 0.001
eGFR (mL/min/1.73 m^2^)	55.23 ± 26.05	70.56 ± 15.48	41.39 ± 26.06	< 0.001
Triglyceride (mmol/L)	1.77 ± 0.85	1.45 ± 0.67	2.05 ± 0.90	0.006
Cholesterol (mmol/L)	4.80 ± 1.34	5.13 ± 1.16	4.65 ± 1.71	0.110
LDL‐C (mmol/L)	2.77 ± 1.10	3.25 ± 0.98	2.33 ± 1.02	< 0.001
HDL‐C (mmol/L)	1.41 ± 0.40	1.56 ± 0.40	1.28 ± 0.37	0.007
ACR (mg/g)	1965.87 ± 1483.18	NA	1965.87 ± 1483.18	

Abbreviations: ACR = urinary albumin‐to‐creatinine ratio; BUN = hemoglobin, blood urea nitrogen; eGFR = estimated glomerular filtration rate; HDL‐C = high‐density lipoprotein cholesterol; LDL‐C = low‐density lipoprotein cholesterol.

### 3.2 DKD Is Associated With Inflammation and Leaky Gut Markers

**FIGURE 1:**
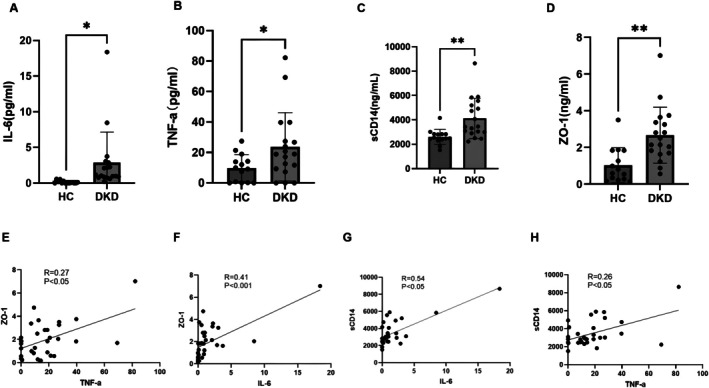
Systemic inflammation was associated with impaired intestinal barrier function in DKD patients. (A, B) Plasma TNF‐α and IL‐6 were analyzed by ELISA measurements. (C, D) Gut barrier function was assessed using ZO‐1 and sCD14 ELISA measurements in plasma. (E–H) Inflammation and leaky gut indicators were associated using Spearman correlation analysis. *p ≤ 0.05; **p ≤ 0.01.

In the DKD group, serum levels of the pro‐inflammatory cytokines interleukin (IL)‐6 and tumor necrosis factor (TNF)‐α were significantly higher than in the HC group (Figure 1A,B). The serum levels of the tight junction protein zonula occludens (ZO)‐1 and the lipopolysaccharide (LPS) binding protein soluble cluster of differentiation (sCD14) were also significantly higher in the DKD group than in the HC group (Figure 1C,D), which indicates the presence of endotoxemia and intestinal barrier dysfunction in the DKD group. Flow cytometry further identified significantly higher circulating levels of natural killer (NK) T cells (1.5‐fold, *p* = 0.015) and CD8+ T cells (1.5‐fold, *p* = 0.018), and a significantly higher lymphocyte ratio (2.4‐fold, *p* = 0.009), in the HC group than in the DKD group. By contrast, CD4+ cells (1.7‐fold, *p* = 0.002), CD4+ central memory T (TCM) cells (1.2‐fold, *p* = 0.042), CD8+ TCM cells (1.6‐fold, *p* = 0.043), the CD4+/CD8+ cell ratio (3.0‐fold, *p* = 0.019), and pro‐inflammatory mediators including IL‐6 (0.13 ± 0.20 pg/mL vs. 2.85 ± 4.31 pg/mL, *p* = 0.03), TNF‐α (9.68 ± 8.92 pg/mL vs. 23.64 ± 22.45 pg/mL, *p* = 0.04), sCD14 (2604.50 ± 607.57 ng/mL vs. 4126.31 ± 1652.01 ng/mL, *p* = 0.001), the gut barrier dysfunction index, and expression of ZO‐1 (1.03 ± 0.96 ng/mL vs. 2.67 ± 1.53 ng/mL, *p* = 0.001), were significantly higher in the DKD group than in the HC group (Figure 1A,B).

Correlation analyses demonstrated significant positive associations between serum ZO‐1 levels and both TNF‐α (*r* = 0.27, *p* < 0.05) and IL‐6 (*r* = 0.41, *p* < 0.001) concentrations (Figure 1E,F). Similarly, sCD14 exhibited strong correlations with TNF‐α (*r* = 0.26, *p* < 0.05) and IL‐6 (*r* = 0.54, *p* < 0.05) levels (Figure 1G,H). These results suggest an interconnection between gut barrier dysfunction and systemic inflammation in DKD pathogenesis.

### 3.3 Gut Microbiota Alterations in Patients With DKD

**FIGURE 2:**
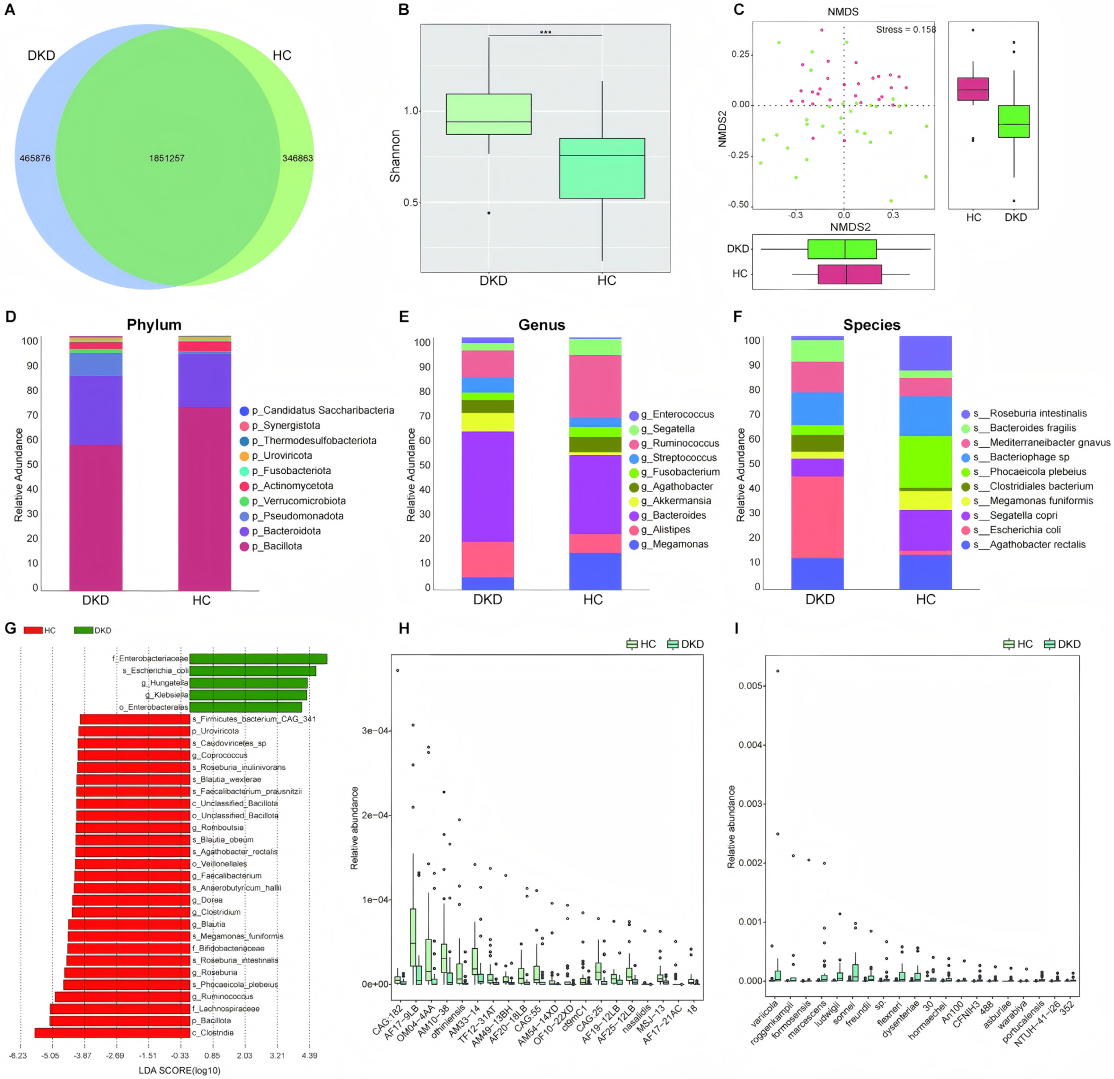
Characteristics of gut microbiota in DKD patients compared to healthy controls. Analysis of gut microbiota between DKD (*n* = 31) and HC (*n* = 28) groups through metagenomic sequencing. (A) Venn diagrams of the unigenes between DKD and HC groups. (B) Difference in the α‐diversity between two groups. (C) β‐diversity analysis between the two groups. (D) Analysis of bacterial composition on phylum level. (E) Analysis of bacterial composition on genus level. (F) Analysis of bacterial composition on species level. (G) Differences in the microbial taxa, as determined by LEfSe coupled with effect size measurements, between the DKD (green) and HC (red) groups. (H, I) Comparing the differences in microbial communities between two groups through MetaStat analysis. Statistical significance was assessed using two‐tailed Student's *t* test. *p ≤ 0.05; **p ≤ 0.01; ***p ≤ 0.001.

Given the potential association between intestinal barrier dysfunction and microbial dysbiosis in DKD, metagenomic sequencing was conducted to characterize gut microbiota composition. Analysis of bacterial composition based on unigene levels revealed significant differences between the two groups (Figure 2A), which were observed in both the taxonomic richness and diversity of the gut microbiota (Figure 2B). The alpha‐diversity of the gut microbiota in the DKD group was significantly higher than that in the HC group (Figure 2B). The beta‐diversity analysis of the two groups was determined by nonmetric multidimensional scaling. The gut microbiota of the DKD group was distinct from that of the HC group, indicating that the DKD group had an altered gut microbiota composition (Figure 2C, *p* < 0.05). Furthermore, we carried out a detailed comparison of the gut microbiota across various classification levels between the two groups. At the phylum level, there was increased abundance of Pseudomonadota and Bacteroidota in the DKD group compared with the HC group (Figure 2D). Meanwhile, at the genus level, the abundance of *Akkermansia*, *Bacteroides*, and *Alistipes* in the DKD group was higher than in the HC group (Figure 2E). Similar results were also found in *Escherichia coli* , *Clostridiales* bacterium, and *Mediterraneibacter gnavus* according to species composition analyses (Figure 2F). Finally, we identified differences in specific bacteria between the DKD and HC groups using Linear discriminant analysis Effect Size and MetaStats analysis, which revealed five bacterial species specific to the DKD group (Figure 2G–I).

In addition, we discovered that the abundance of *Enterobacter roggenkampii*, *Serratia marcescens* , uncultured *Ruthenibacterium* sp., *Shigella flexneri* , *Shigella dysenteriae* , *Enterobacter ludwigii* , *Shigella sonnei* , *Citrobacter freundii* , and *Enterobacter hormaechei* was higher in the DKD group than in the HC group (Figure S1). All these observations suggest that patients with DKD have an altered gut microbiota composition compared with HC, and that alterations in the gut microbiota may be attributed to the progression of DKD.

### 3.4 Immune Cell Alterations in Patients With DKD

**FIGURE 3:**
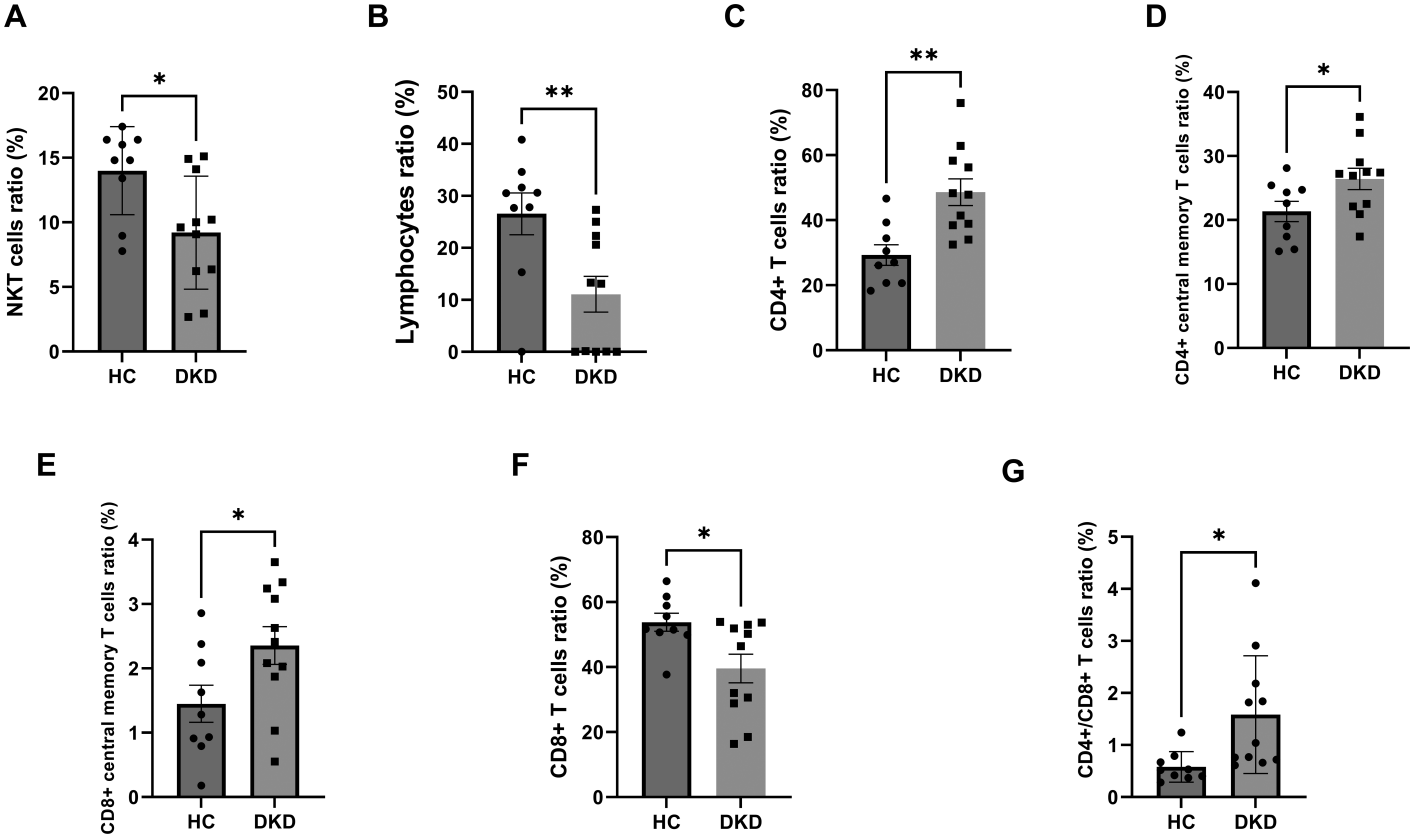
Immune cells alterations in DKD patients compared to healthy controls. Analysis of changes in relevant immune cell populations including NKT cells (A), lymphocytes (B), CD4+ T cells (C), CD4+ central memory T (TCM) cells (D), CD8+ TCM cells (E), CD8+ T cells (F) and CD4+/CD8+ cells ratio (G) in DKD patients (*n* = 11) and healthy people (*n* = 9) through flow cytometry. *p ≤ 0.05, **p ≤ 0.01.

To gain a comprehensive understanding of changes in relevant immune cell populations in DKD, we collected peripheral blood from HC (*n* = 9) and patients with DKD (*n* = 11) for immunophenotyping by flow cytometry. Compared with the HC group, the DKD group exhibited significantly higher levels of CD4+ T cells, the CD4+/CD8+ cell ratio, CD4+ TCM cells (CD3+CD4+CD45RA−CCR7+), and CD8+ TCM cells (CD3+CD8+CD45RA−CCR7+) (all *p* < 0.05). In addition, the percentages of NKT cells (CD3+CD56+) and the lymphocyte ratio were significantly lower in the DKD group than in the HC group (both *p* < 0.05) (Figure 3, Figure S2). By contrast, no statistically significant differences were observed in the levels of plasmacytoid dendritic cells (LIN−CD11B−HLADR+), granulocytes, macrophages, classic dendritic cells (LIN−CD11B+CD15+CD16+CD68−), neutrophils (LIN−CD11B+CD15+CD16+), B cells (CD3−CD56−CD19+), NK cells (CD3−CD56+), or γδ T cells (CD3+CD56−TCRγδ+) between the two groups (all *p* > 0.05) (Figure S3).

**FIGURE 4:**
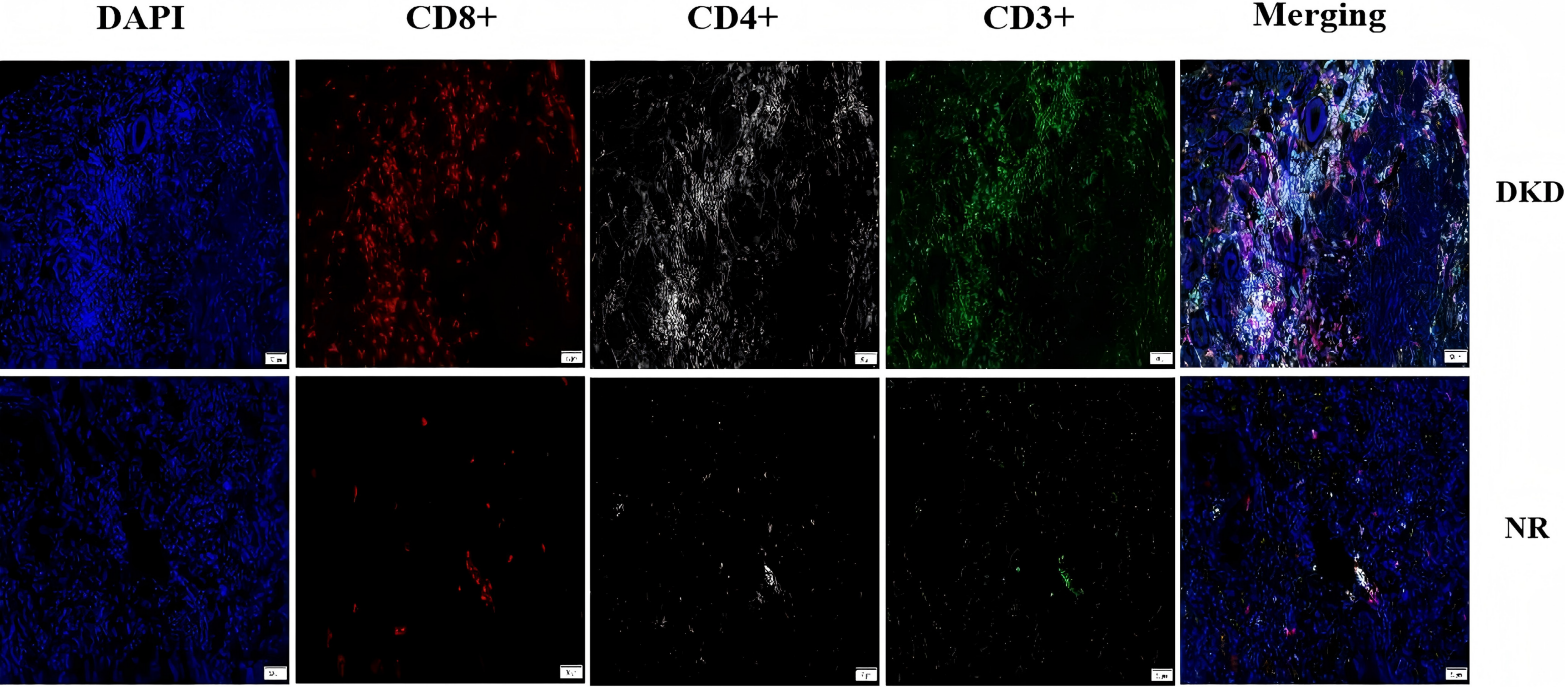
Immune cell alterations in DKD patients compared to the normal renal tissue (NR). Definition of normal renal tissue: Renal parenchyma located at a distance of ≥ 2 cm from the margin of renal cell carcinoma (RCC), confirmed as histopathologically normal by pathological examination.

Furthermore, we analyzed immune cells in the pathological tissues of patients with DKD and the normal renal tissue (NR) and found more CD3+, CD4+, and CD8+ cells in DKD tissues (Figure 4). Together, these results indicate that dysregulation of specific T cell subsets, rather than broad immune shifts, may contribute to DKD pathogenesis and represent potential immunological targets for future therapeutic intervention.

## 4 Discussion

DKD is a serious kidney disease and a major complication of diabetes. The pathogenesis of DKD is complex and usually influenced by multiple factors. The gut microbiota has recently emerged as a significant contributor to the development and progression of DKD. In this study, we utilized metagenomic sequencing to analyze the structure and functional characteristics of the gut microbiota in patients with DKD. We observed significant differences in the diversity and richness of gut microbiota between patients with DKD and HC; specifically, the relative abundance of Enterobacteriaceae, *Serratia*, and *Shigella* was significantly higher in patients with DKD. Therefore, the functional composition of the gut microbiota showed obvious alterations, which might influence the progression of DKD. We also found that CD3+ T cells, especially CD4+ cells, were significantly higher in the renal tissue of patients with DKD than in that of control patients. In addition, CD3+ T cells, CD4+ T cells, the CD4+/CD8+ T cell ratio, NKT cells, the lymphocyte ratio, IL‐6, TNF‐α, sCD14, and ZO‐1 were also significantly higher in the serum of patients with DKD compared with HC.

Growing data suggest that gut dysbiosis plays a crucial role in the development of chronic systemic inflammation [12]. The potent immunostimulant LPS, which is found on the outer membrane of gram‐negative bacteria, has been linked to inflammatory markers such as TNF‐α, IL‐6, and C‐reactive protein, all of which exhibit increased abundance in the gut of patients with DKD [13]. The development of bacteria with proteolytic properties that promote the production of the uremic toxins indoxyl sulfate, p‐cresyl sulfate, indole‐3‐acetic acid, and trimethylamine N‐oxide is also responsible for triggering inflammatory and oxidative stress responses [14]. Furthermore, the development of urease‐producing bacteria in patients with DKD raises the pH and ammonia concentrations in the gut lumen, which in turn causes mucosal irritation and increases intestinal permeability [15, 16]. In our study, patients with DKD exhibited significant intestinal barrier dysfunction, which was manifested by elevated serum ZO‐1 and sCD14 levels. ZO‐1 regulates intestinal permeability; it significantly increases permeability and impairs barrier function in various situations, such as obesity, diabetes, and autoimmune diseases [17, 18, 19, 20]. Impaired barrier function allows the translocation of luminal LPS into the systemic circulation. In line with this mechanism, we detected significantly higher serum levels of the LPS‐binding protein sCD14 in patients with DKD, indicating that LPS may induce inflammation in this population. Indeed, elevated serum sCD14 levels have been associated with an increased risk of two independent cardiovascular diseases. However, intestinal barrier dysfunction may be either a cause or an effect of microbial metabolite imbalance [21, 22]. Therefore, we analyzed the composition of the gut microbiota in patients with DKD and HC.

DKD is a disease related to a variety of immune factors [23, 24]; however, inflammation and immune system activation are two of the most important mechanisms in its progression. In this study, we found that CD3+ T cells, especially CD4+ T cells, were significantly higher in the renal tissue of patients with DKD than in that of control patients. CD4+ T cells, CD4+ TCM cells, CD8+ TCM cells, and the CD4+/CD8+ cell ratio were significantly higher in the serum of patients with DKD than in that of HC. The increase in these immune cells may be due to an inflammatory response in the body, either as part of the systemic inflammation that can contribute to DKD progression or a compensatory response in DKD aimed at regulating renal inflammation. Either way, an excessive immune response may exacerbate tissue damage and accelerate the development of DKD. Therefore, monitoring the immune status of patients with DKD is essential to assess disease progression and guide therapeutic strategies.

The correlation between gut microbiota diversity and DKD is currently controversial because of inconsistent findings [25, 26, 27]. For instance, Zhang et al. demonstrated that there was no significant difference in the diversity of the gut microbiota between control and DKD groups [1]. However, we observed significant differences in the diversity and richness of the gut microbiota between patients with DKD and HC, with a significantly increased abundance of Enterobacteriaceae, *Serratia*, and *Shigella* in the DKD group. These differences may be related to detection methods, metabolic status, and drug use. Our results suggest that the balance of intestinal flora in patients with DKD was disrupted, specifically because of a reduced abundance of beneficial bacteria such as *Lactobacilli*, and an enrichment of pathogenic bacteria including *Proteus*, *E. coli* , and *Shigella*. These alterations of the gut microbiota are often associated with increased intestinal permeability, systemic inflammation, and metabolic dysfunction, all hallmarks of DKD pathogenesis. Furthermore, the decline in glomerular filtration rate associated with DKD impairs renal excretion, leading to the accumulation of metabolic waste products in the body. These metabolic waste products can translocate into the intestinal cavity through the intestinal wall, further altering the gut environment and aggravating gut microbiota dysbiosis. Additionally, metabolic abnormalities and chronic inflammation in patients with DKD affect gut microbiota composition. Chronic inflammation activates immune cells to release inflammatory cytokines, which further damage the intestinal barrier and increase the proportion of harmful bacteria. Reduced levels of microbial metabolites such as short‐chain fatty acids, along with the accumulation of uremic toxins, can also lead to intestinal mucosal damage and dysbiosis, which results in a significant enrichment of harmful bacteria such as Enterobacteriaceae [28, 29]. In short, the disruption of gut microbiota homeostasis in patients with DKD leads to an increased abundance of pathogenic bacteria such as Enterobacteriaceae, *Serratia*, and *Shigella*, which worsens disease progression and amplifies systemic inflammation. Collectively, these findings suggest that gut microbiota dysbiosis in DKD may not only reflect disease progression but also contribute to it through immune modulation, the production of uremic toxins, and the disruption of intestinal barrier integrity. The identification of these specific bacterial alterations provides potential biomarkers for DKD and lays the groundwork for microbiota‐targeting therapeutic strategies.

There are some limitations to this study. First, the conclusions should be interpreted with caution because of the relatively small sample size. Second, additional clinical indices are needed to more accurately assess the role of the gut microbiota in DKD. Lastly, it is well established that medications can influence the gut microbiota composition. Most participants in this study took hypoglycemic or antihypertensive drugs, which may have affected their microbial profiles.

In summary, this study provides insight into the composition and functional characteristics of the gut microbiota in patients with DKD and HC through metagenomic sequencing analysis. Our results suggest that the gut microbiota plays a critical role in DKD pathogenesis, with inflammation and immune system activation being key mechanisms in its progression. Nevertheless, more studies are warranted to clarify the precise role of the gut microbiota in metabolic processes. Future research should aim to identify novel diagnostic and therapeutic strategies for DKD that target the gut microbiota.

## Author Contributions

**Xueting Zheng:** writing – review and editing, writing – original draft, funding acquisition. **Xiaoyan Luo:** review and editing, writing – original draft. **Yi Zhang:** writing – review and editing, writing – original draft. **Zhiyan Zou:** software, methodology. **Jiayi Yang:** formal analysis. **Huan Liu:** validation. **Zhou Lu:** methodology. **Fangfang Cao:** visualization, software. **Xilian Wang:** visualization, software. **Xinyun Ge:** software, methodology. **Xiaoan Li:** supervision, funding acquisition, conceptualization. **Jiali Wang:** supervision, funding acquisition, conceptualization.

## Funding

This work was supported by National Clinical Key Specialty Research Projects in Gastroenterology (Grant No. XHZDZK019), Incubation Project of Mianyang Central Hospital (Grant No. 2020FH09), NHC Key Laboratory of Nuclear Technology Medical Transformation (Mianyang Central Hospital, No. 2022HYX005), The Sichuan Science and Technology Program (Grant No. 2023YFS0470), Mianyang Science and Technology Bureau (Mianyang Science and Technology Program, Grant No. 2023ZYDF073).

## Ethics Statement

This study were approved by the Biomedical ethics committee of Mianyang Central Hospital (No. S20240323‐01).

## Conflicts of Interest

The authors declare no conflicts of interest.

## Supporting information


**Figure S1:** The significantly different species between HC and DKD group. Nine bacteria showed a significant difference at the species level between DKD patients (*n* = 31) and HC individuals (*n* = 28). Statistical significance was denoted as follows: *p ≤ 0.05; **p ≤ 0.01; ***p ≤ 0.001.

**Figure S2:** Flow clustering diagram between DKD patients and healthy controls people. (A) NKT cell; (B) lymphocytes; (C): CD4; CD8 cell; (D) CD4+ TCM cells; (E) CD8+ TCM cells.

**Figure S3:** Immune cells alterations in DKD patients compared to healthy controls. Analysis of changes in relevant immune cell populations in DKD patients (*n* = 11) and healthy people (*n* = 9) through flow cytometry. ns, *p* > 0.05.

## Data Availability

The data that supports the findings of this study are available in the Supporting Information of this article.
