# Supplementary figures and images for: Inflammation in Diabetic Kidney Disease Is Linked to Gut Dysbiosis and Metabolite Imbalance
Source: J Diabetes. 2025 Dec 14;17(12):e70175. doi: 10.1111/1753-0407.70175 (PMC12702812; doi:10.1111/1753-0407.70175)

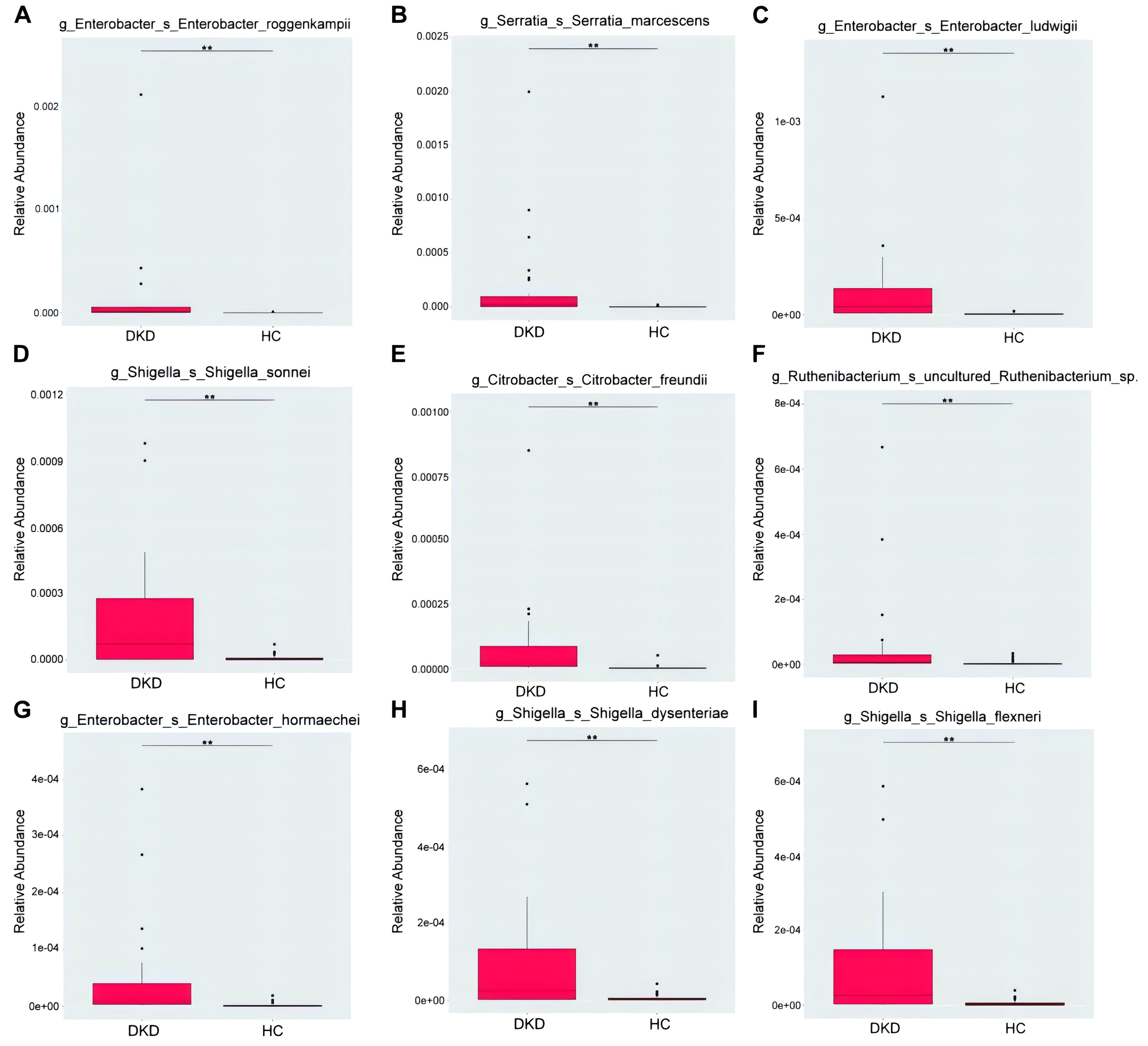

Supplement: Supplementary file 1 — Figure S1: The significantly different species between HC and DKD group. Nine bacteria showed a significant difference at the species level between DKD patients (n = 31) and HC individuals (n = 28). Statistical significance was denoted as follows: *p ≤ 0.05; **p ≤ 0.01; ***p ≤ 0.001. [file JDB-17-e70175-s003.tif]

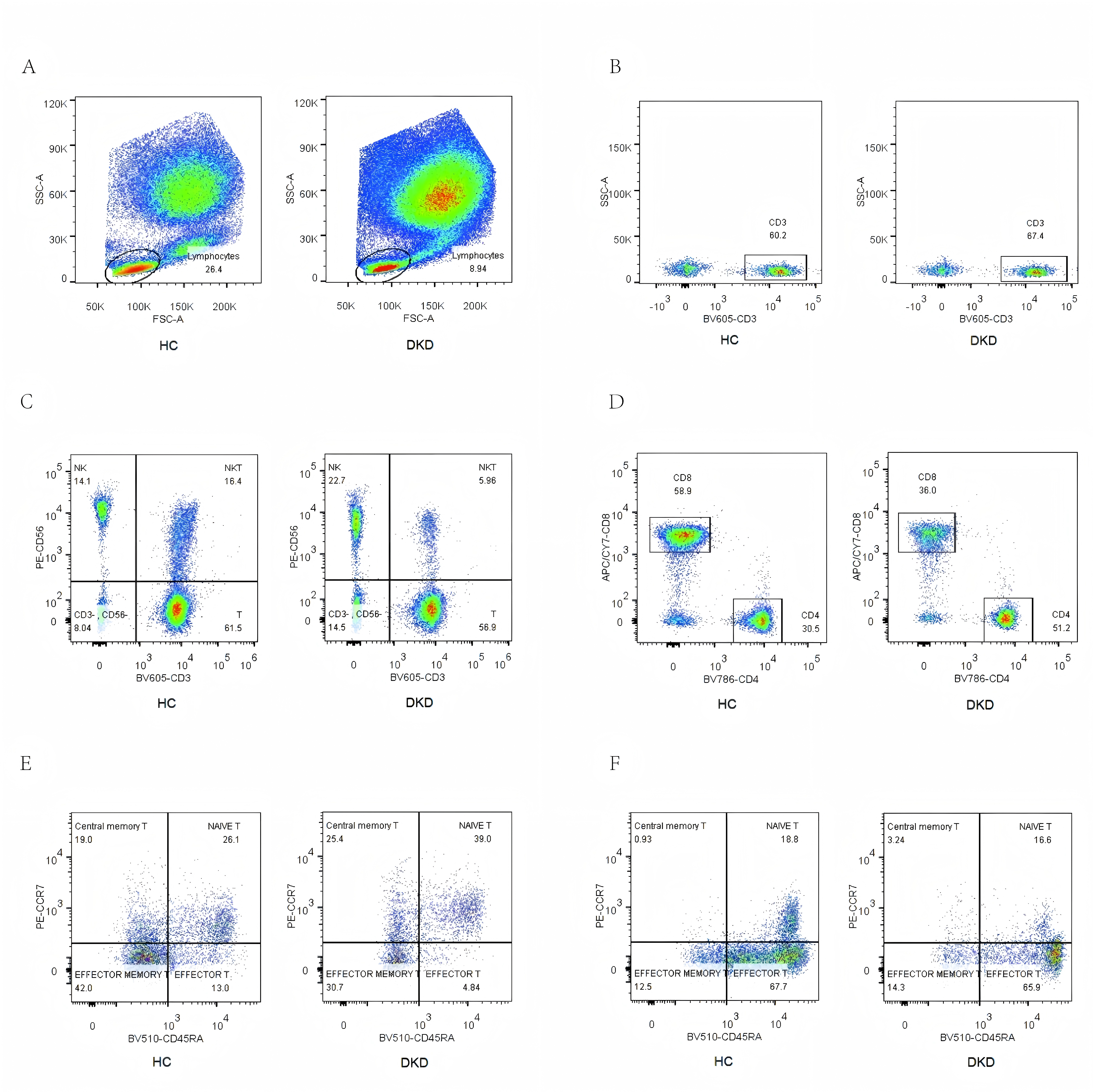

Supplement: Supplementary file 2 — Figure S2: Flow clustering diagram between DKD patients and healthy controls people. (A) NKT cell; (B) lymphocytes; (C): CD4; CD8 cell; (D) CD4+ TCM cells; (E) CD8+ TCM cells. [file JDB-17-e70175-s002.tif]

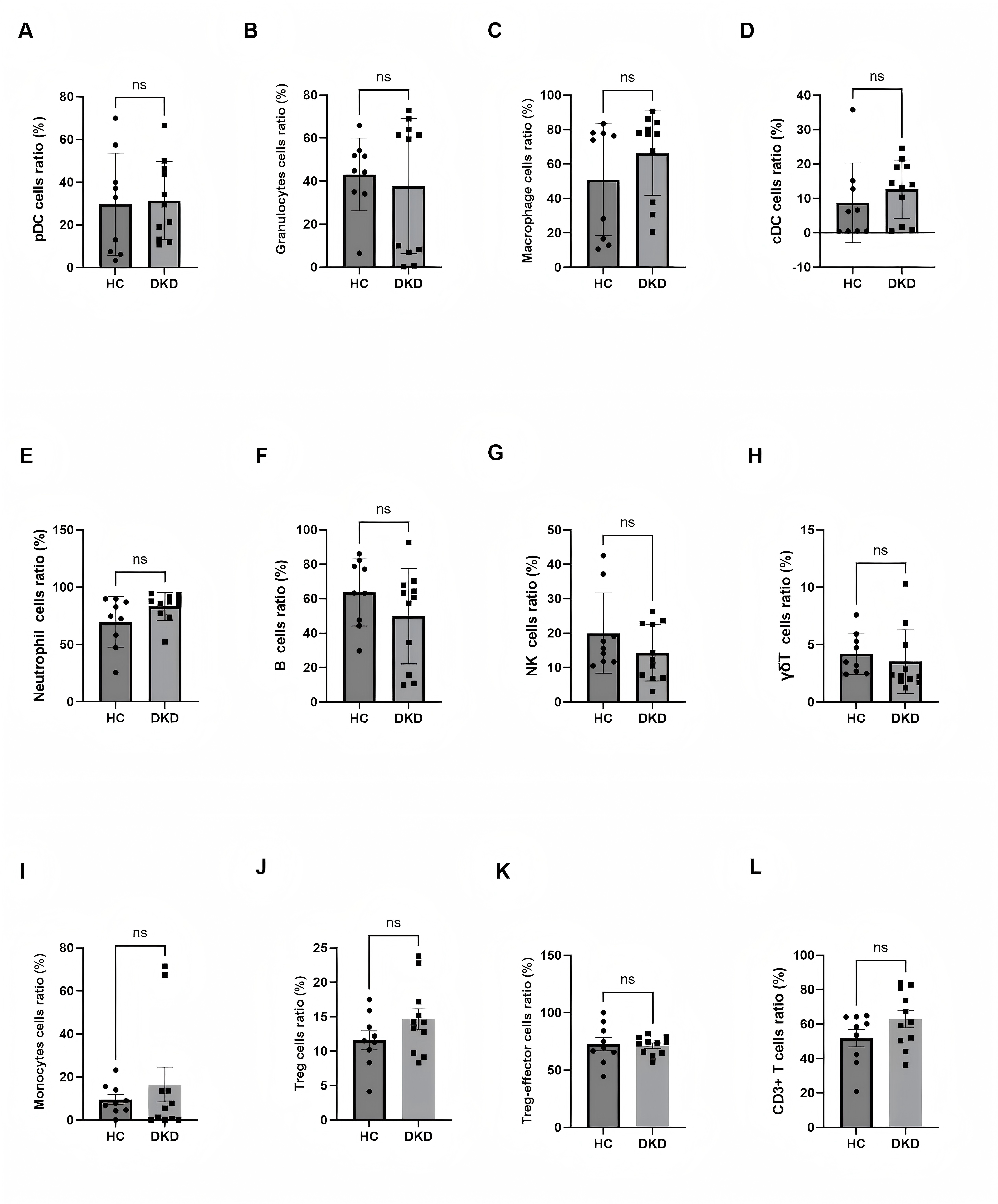

Supplement: Supplementary file 3 — Figure S3: Immune cells alterations in DKD patients compared to healthy controls. Analysis of changes in relevant immune cell populations in DKD patients (n = 11) and healthy people (n = 9) through flow cytometry. ns, p > 0.05. [file JDB-17-e70175-s001.tif]
